# *Agrobacterium tumefaciens*-Mediated Transformation of *Pseudocercospora fijiensis* to Determine the Role of *PfHog1* in Osmotic Stress Regulation and Virulence Modulation

**DOI:** 10.3389/fmicb.2017.00830

**Published:** 2017-05-16

**Authors:** Francis Onyilo, Geoffrey Tusiime, Li-Hung Chen, Bryce Falk, Ioannis Stergiopoulos, Jaindra N. Tripathi, Wilberforce Tushemereirwe, Jerome Kubiriba, Charles Changa, Leena Tripathi

**Affiliations:** ^1^National Agricultural Research LaboratoriesKampala, Uganda; ^2^Department of Agricultural Production, College of Agricultural and Environmental Sciences, Makerere UniversityKampala, Uganda; ^3^International Institute of Tropical AgricultureNairobi, Kenya; ^4^Department of Plant Pathology, University of California, Davis, DavisCA, USA

**Keywords:** *Agrobacterium tumefaciens*, transformation, *Pseudocercospora fijiensis*, *HOG1*, osmotic stress, virulence

## Abstract

Black Sigatoka disease, caused by *Pseudocercospora fijiensis* is a serious constraint to banana production worldwide. The disease continues to spread in new ecological niches and there is an urgent need to develop strategies for its control. The high osmolarity glycerol (HOG) pathway in *Saccharomyces cerevisiae* is well known to respond to changes in external osmolarity. HOG pathway activation leads to phosphorylation, activation and nuclear transduction of the HOG1 mitogen-activated protein kinases (MAPKs). The activated HOG1 triggers several responses to osmotic stress, including up or down regulation of different genes, regulation of protein translation, adjustments to cell cycle progression and synthesis of osmolyte glycerol. This study investigated the role of the MAPK-encoding *PfHog1* gene on osmotic stress adaptation and virulence of *P. fijie*nsis. RNA interference-mediated gene silencing of *PfHog1* significantly suppressed growth of *P. fijiensis* on potato dextrose agar media supplemented with 1 M NaCl, indicating that *PfHog1* regulates osmotic stress. In addition, virulence of the *PfHog1*-silenced mutants of *P. fijiensis* on banana was significantly reduced, as observed from the low rates of necrosis and disease development on the infected leaves. Staining with lacto phenol cotton blue further confirmed the impaired mycelial growth of the *PfHog1* in the infected leaf tissues, which was further confirmed with quantification of the fungal biomass using absolute- quantitative PCR. Collectively, these findings demonstrate that *PfHog1* plays a critical role in osmotic stress regulation and virulence of *P. fijiensis* on its host banana. Thus, *PfHog1* could be an interesting target for the control of black Sigatoka disease in banana.

## Introduction

Banana and plantain (*Musa* sp.) is the eighth most economically staple food crop (Tripathi et al., 2014). Worldwide it is cultivated across tropical and subtropical countries on more than 10 million hectares and total production estimated at 144 million tons (FAOSTAT, 2013). It is one of the fundamental energy sources for millions of people in developing countries and the most significant of all fruits, with world trade total of $2.5 billion annually. It is mainly grown by small holder farmers and only 10% of the annual output reaches the international market (Ploetz, 2001).

In Africa, banana and plantain are mainly used as a staple food, providing more than 25% of the carbohydrate requirements for over 70 million people (IITA, 1998). It is also a critical and often the only source of income for both small and large-scale farmers, thus being an important contributor to local economies. The Great Lakes region including Burundi, Rwanda, Democratic Republic of Congo, Uganda, Kenya and Tanzania is the largest producer and consumer of banana in Africa (Smale et al., 2006), with Uganda ranked the third largest producer of the crop worldwide and largest producer in Africa. Although banana is the most important staple food crop in Uganda, its production is declining since 2010 (FAOSTAT, 2013), probably due to pests and diseases.

The ascomycete fungus *Pseudocercospora fijiensis* (synonym *Mycosphaerella fijiensis*) is the causal agent of black Sigatoka disease, one of the important diseases of banana worldwide (Arango Isaza et al., 2016). Black Sigatoka disease can reduce yields by 33 to 76% if left uncontrolled (Gauhl et al., 2000; Marin et al., 2003; Agrios, 2005). The fungus induces necrotic leaf streak and lesion symptoms, which later coalesce into blotches that can cover over 70% of the banana leaf lamina. This significantly reduces the photosynthetic capacity of plants, resulting into poor quality fruits and often premature ripening (Marin et al., 2003). *P. fijiensis* produces conidia and ascospores, and both can cause disease. They are disseminated by wind, and in the case of conidia, spread can also be by water. Ascospores are more important than conidia in spreading the disease within banana plants and plantations. In developing countries, infected planting material and leaves, which are often used as packing materials, are also responsible for the long-distance spread of the disease (Ploetz, 2001).

Mitogen-activated protein kinases (MAPKs) are conserved eukaryotic serine/threonine protein kinases with vital roles in numerous cellular processes, including gene expression, cell differentiation, mitosis, cell survival and apoptosis. Additionally, MAPKs partake in signal transduction pathways that are activated by osmotic stress and in regulation of growth and development (Alonso-Monge et al., 2001). The high osmolarity glycerol (HOG) pathway in *Saccharomyces cerevisiae* is well known to respond to changes in external osmolarity. HOG pathway activation leads to phosphorylation, activation and nuclear transduction of the HOG1 MAPK. The activation of HOG1 triggers several responses to osmotic stress, including up or down regulation of different genes, regulation of protein translation, adjustments to cell cycle progression and synthesis of osmolyte glycerol (Hohmann, 2002).

The HOG MAPKs pathway signaling cascade in fungi regulates responses to stress and adaptation to hyperosmotic conditions (Alonso-Monge et al., 2001). For example, HOG1 pathway was found critical in regulating colonization of mouse gastrointestinal tract in *Candida albicans* (Prieto et al., 2014). In the entomopathogenic fungus *Metarhizium acridum*, *MaHog1*, a member of the *Hog1/Sty1/*p38 MAP-kinase gene family, has been shown to be critical for adaptation to hyper osmolarity, high temperature and oxidative stress (Jin et al., 2012). It is also an important virulence factor as disruption of *MaHog1* results in reduced infectivity and growth of the fungus on its insect hosts (Jin et al., 2012). In a similar way, *Hog1* is also activated in response to high osmolarity, oxidative stress, and other stress stimuli in *S. cerevisiae* and the human pathogenic yeast *C. albicans* (Alonso-Monge et al., 2001; Winkler et al., 2002), whereas in the wheat pathogen *Zymoseptoria tritici* (synonym *M. graminicola*), *Hog1* regulates dimorphism and pathogenicity (Mehrabi et al., 2006). Given the importance of *Hog1* for virulence in fungi and the relatively close phylogenetic relatedness between *Z. tritici* and *P. fijiensis*, it is possible that *PfHog1* is also an important virulence factor for causing black Sigatoka disease.

Gene specific silencing by RNA interference (RNAi) has been widely used to understand functional genomics of fungi over the last couple of decades (Agrawal et al., 2003). In addition, RNAi can be used in control of fungal diseases in plants through host induced gene silencing (HIGs) (Nowara et al., 2010). Proof of concept for HIGS in plant pathogenic fungi was obtained for barley powdery mildew caused by *Blumeria graminis* and *Fusarium verticillioides* (Nowara et al., 2010). Transgenic wheat expressing an RNAi hairpin construct targeting *β-1, 3-glucan synthase* gene Fc*Gls1* of *F. culmorum*, showed enhanced resistance to Fusarium head blight disease (Chen et al., 2016). Similarly, host induced post-transcriptional mediated gene silencing of the fungal genes, *velvet* and *F. transcriptional factor 1*, showed resistance against *F. oxysporum* f sp. *cubense* (Foc) in transgenic banana (Ghag et al., 2014).

The aim of this study was to investigate the role of *PfHog1* in adaptation to osmotic stress and virulence of *P. fijiensis* on its host banana. Silencing of *PfHog1* through *Agrobacterium tumefaciens*-mediated transformation (ATMT) was tested for osmotic stress and for virulence on non-transgenic tissue culture banana plantlets. Virulence was confirmed by visual necrosis, staining with lacto phenol cotton blue (LPCB) and absolute quantitation by qPCR. This study confirmed that silencing of *PfHog1* limits *P. fijiensis* adaptation to osmotic stress and virulence.

## Materials and Methods

### Isolation of *P. fijiensis*

*Pseudocercospora fijiensis* was isolated from infected leaves of banana cultivar ‘Nakitembe’. The infected leaves were collected from the field, cut into pieces of 5 cm and then placed in a moist cotton wool inside a clear polythene bag. The leaf culture was incubated at 25°C overnight, in order to allow maturation of spores. The leaf pieces were recovered, cleaned with 70% ethanol and then sterile water. The mature conidia were collected onto 1% agar (w/v in water) by stapling necrotic leaves under moist blot paper in a petri- dish and incubated for 24 h at 25°C. The conidia discharged on agar were picked under a light microscope using a sterile needle and transferred to V8 juice agar medium [30% (v/v) V8 juice, 3 g CaCo3, 20 g bacterial agar, pH 7.2]. Cultures were incubated at 25°C for 14 days; the resulting pure isolates of *P. fijiensis* were then stored at room temperature.

### Isolation of Genomic DNA of *P. fijiensis* and PCR Amplification of *PfHOG1* Gene

Genomic DNA of *P. fijiensis* was extracted from the pure culture mycelia as described (Mahuku, 2004) with minor modification, which involved incubation of the sample at room temperature for a period of 12–13 h and then overnight at -20°C.

*PfHog1* gene of *P. fijiensis* was amplified from the genomic DNA extract using *PfHog1* specific primers [HOG1F: ACGGAGCTGCGTAACGAATTAG and HOG1R: CTGCGTGTGATCGACTAG]. The primers were designed based on *P. fijiensis* CIRAD86 MAP kinase sequences (accession number XM_007924474.1). The PCR reaction mixture contained 10 μM each of forward and reverse primers (0.5 μl), AmpliTaq^®^ DNA polymerase (0.25 μl; Applied Biosystems, USA), 10x Buffer with 15 mM MgCl2 (2.5 μl), (Applied Biosystems, USA), 10 μM deoxyribonucleotides (dNTP; 0.5 μl), 1 μl of genomic DNA of *P. fijiensis* and then adjusted with water to 25 μl final volume. The cycle conditions used were the following: initial denaturation at 95°C for 5 min and then 34 cycles of denaturation at 95°C for 30 s, annealing at 55°C for 30 s and extension at 72°C for 1 min, followed by final extension at 72°C for 5 min and storage at 12°C. The amplified PCR product was separated by electrophoresis on agarose gel.

### Preparation of Plasmid Construct

**FIGURE 1:**
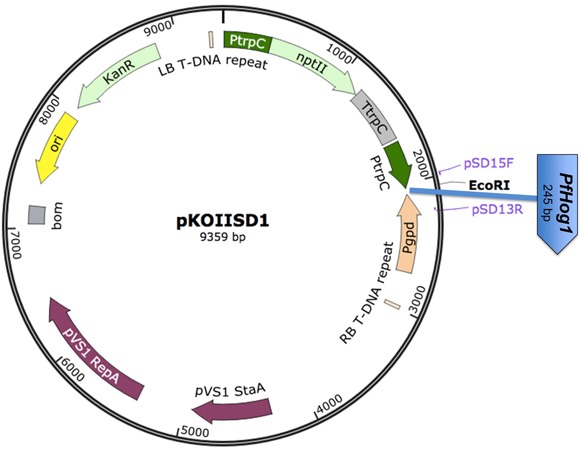
**Schematic diagram of the RNAi plasmid construct pKOIISD1-*PfHog1*.** PtrpC, promoter *trpC* of *Aspergillus nidulans*; Pgpd, promoter *gpd* of *A. nidulans*; *npt*II, neomycin phosphotransferase II gene; *PfHog1*, *Hog1* gene from *P. fijiensis;* LB- Left Border of T-DNA; RB- Right Border of T- DNA fragment.

The amplified *PfHog1* product was resolved by electrophoresis on 1% agarose gel and then purified by Zymoclean^TM^ Gel DNA recovery kit following the manufacturer’s protocol. The purified DNA was ligated into pGEM^®^-T easy vector according to the procedure in the manual of Promega, Madison, W1, USA, and incubated at 16°C overnight. The ligated vector was later digested with EcoRI. The product *PfHog1* was ligated into RNAi silencing pKOIISD1 fungal transformation vector at EcoRI site (**Figure 1**). The pKOIISD1 was designed based on pSilent-Dual1 (pSD1) vector with dual promoters (i.e., sense promoter PtrpC and antisense promoter Pgpd), provided by Stergiopoulos laboratory at University of California Davis, USA. Basically, pKOIISD1 is the combination of pSD1 and pBHt2 vectors. Three restriction enzyme sites (ApaI, BglII, and AvrII) were inserted into XmnI site of pBHt2. The pSD1 was digested with ApaI and SacI to obtain DNA fragment containing geneticin-resistant marker, promoter PtrpC and promoter Pgpd. Subsequently, this DNA fragment was ligated into ApaI/SacI digested pBHt2 to generate pKOIISD1.

The pKOIISD1-*PfHog1* plasmid was validated by PCR and sequencing for the presence and orientation of insert. After validation the plasmid was transformed into *Escherichia coli* strain DHα5 according to high efficiency transformation protocol of the New England Biolabs.

### Transformation of *P. fijiensis*

#### Culturing of *P. fijiensis*

A mycelial plug of *P. fijiensis* was grinded and the fungal suspension (10 ml) added to 200 ml of sterile rich medium (2% yeast and 3% glucose in water) supplemented with 100 μg/ml ampicillin and 100 μg/ml cefotaxim in a flask. The culture was then incubated at 25°C with gentle shaking at 200 rpm for 5 to 7 days at room temperature.

#### Transformation of *Agrobacterium tumefaciens*

The electro-competent *A. tumefaciens* strain AGL1 (40 μl) was dispensed in eppendorf tube and 5 μl pKOIISD1-*PfHog1* (500 ng/μl) plasmid DNA was added. The mixture was then electroporated by applying an electric pulse (2.5 kV), capacitance (25 μF) and resistance (400 Ω). Immediately LB medium (300 μl) was added to the transformed cells followed by incubation at 28°C with shaking at 230 rpm for 4 h. Then 60 and 100 μl of the culture were plated on LB agar supplemented with 50 μg/ml kanamycin and incubated at 28°C for 2 days. Single colonies were picked and inoculated into LB broth (10 ml) supplemented with rifampicin and kanamycin at 10 and 50 μg/ml, respectively. The culture was incubated at 25°C with shaking at 230 rpm for 48 h. Thereafter, cells were harvested by centrifugation at 6000 rpm for 10 min. The pellet obtained was re-suspended in 20 ml *Agrobacterium* induction medium (AIM) and acetosyringone (Sigma Aldrich) was added to 200 μM final concentration. The culture was incubated at 25°C with shaking at 230 rpm for 3 h. The *Agrobacterium* culture with an O.D_600__nm_ of 0.2–0.5 were used to transform *P. fijiensis*.

#### Co-cultivation and Selection of Transformed *P. fijiensis*

For infection, the liquid culture of *P. fijiensis* mycelium (200 μl) was mixed with *Agrobacterium* culture (300 μl) and incubated at room temperature for 10min. Thereafter, an aliquot of the mixture (200 μl) was spread onto *Agrobacterium* induction agar media plates covered with cellulose membranes and co-cultivated at 25°C for 3 days. The co-cultivated cultures were transferred to standard potato dextrose agar (PDA) medium supplemented with 150 μg/ml geneticin (G148) and 100 μg/ml cefotaxime. After 5 days, single colonies of *Agro*-infected *P. fijiensis* were transferred to PDA medium containing geneticin (150 μg/ml).

All the transformants of *P. fijiensis* were generated using one plasmid construct pKOIISD1-*PfHog1*, in which the sense strand of *PfHog1* gene was driven by PtrpC promoter and anti-sense strand by Pgpd promoter (**Figure 1**).

### Molecular Characterisation of Transformed *P. fijiensis*

#### Extraction of Genomic DNA and PCR Analysis of Transformed *P. fijiensis*

A plug of transformed mycelia was freeze-dried and then lyophilised. The genomic DNA was extracted as previously described (Thon et al., 2000). The primers, pSD15F (CTTTAAGTTCGCCCTTCCTC), pSD13R (GTTGACAAGGTCGTTGCGT), designed from pKOIISD1 vector were used to amplify *PfHog1* gene from genomic DNA of transformed *P. fijiensis*. PCR reaction mixture contained 10 μM primers pSD15F and pSD13R (0.5 μl each). The PCR reaction component and PCR conditions are same as described above.

After validation by PCR, three mutants of *P. fijiensis* (ΔPfHog1-1, ΔPfHog1-4, and ΔPfHog1-5) were selected randomly from independent transformation experiments for further analysis. All the mutants were generated using same plasmid construct.

### Assessment of Gene Expression in Transformed *P. fijiensis*

#### RNA Extraction and Complementary DNA (cDNA) Synthesis

The wild type (WT) and transformed *P. fijiensis* were grown on V8 juice agar medium at 25°C and total RNA was isolated using Trizol^®^ reagent according to the protocol provided by Ambion RNA life technologies. The RNA extract was further purified by cleaning using RNA clean and concentrator-^TM^ kit following the protocol from Zymo research Corp. Then cDNA was synthesized using Maxima first strand cDNA kit for RT-qPCR, Thermo Fishers Scientific, Inc.

#### Quantitative Real Time-Polymerase Chain Reaction (qRT-PCR) Assay

The qRT-PCR assay was performed using Applied Biosystem 7500 Life Technology machine. Specific primers (TUBF1-ATACACACCGCATCAACGAC and TUBR1-ATGAACGATCTCGCATTC) with amplification product of 114 bp were designed based on *β-tubulin* gene sequence from *P. fijiensis* genome. To detect the level of transcript for silencing in *P. fijiensis*, *PfHog1* gene specific primers (HOGF- TGAAAACGGAGCTGCGTAAC and HOGR-TTCTCACGGTTCCGTAATGC) were designed based on *P. fijiensis* sequence.

A standard curve was made for determining primer specificity, efficiency and for calculation of *P. fijiensis* DNA and Biomass. A known serial dilution of DNA/cDNA was generated with dilution factor of 1/10. The standard curve was then generated using GraphPad Prism software version 5 and Microsoft Excel 2007.

Quantitation comparative *C*_t_ was used in *PfHog1* expression or detection level of transcript for silencing in *P. fijiensis*. The qRT-PCR reaction mixture contained Maxima SYBR Green/ ROX qPCR Master mix (2x; Thermo Scientific), 300 nM primer (i.e., *β- tubulin and Hog1*), 100 ng/μl cDNA in a total reaction volume of 12 μl. RT- PCR program used was as follows; 40 cycles at 50°C for 2 min, 95°C for 10 min, 95°C for 15 s, 60°C for 30 s, and 72°C for 30 s, followed by melting curve stages.

Three different mutants (Δ*PfHog1-1*, Δ*PfHog1-4*, and Δ*PfHog1-5*) and WT control were used with three technical replicates in each experiment. The experiment was repeated three times.

### Osmotic Stress Assay

Freshly grown transformed mycelia were ground and filtered using double layer cheese-cloth under sterile conditions. The mycelia were counted using a haemocytometer and suspension was adjusted to a density of 10^4^–10^5^/ml. About 10^4^–10^5^ mycelia were then used for culturing on each plate. Mycelium mixture was prepared in deionised sterile water in a total volume of 20 ml and 300 μl of the mixture was spread onto PDA medium supplemented with 1 M NaCl. This culture was incubated at 25°C for approximately 10 days and germination of colonies was assessed visually and photographs taken with an ordinary digital camera.

### Virulence Assay

#### Preparation of Inoculums

Freshly ground transformed and non-transformed mycelium fragments was resuspended in 200 ml of rich medium supplemented with 100 μg/ml ampicillin and culture was incubated at room temperature with shaking at 150 rpm for 5–10 days. The fungal culture was filtered using double layer cheese-cloth and mycelium was counted. Then 10^4^–10^5^ mycelia were used for inoculation of each sample. Inoculums were prepared in 10% rich medium containing 1% Tween 20.

#### Inoculation of Banana Plants

Three months old potted tissue culture banana plants were used for virulence assay. Plants were placed in a locally made inoculation chamber in a contained glasshouse. Three leaves per plant of three replicated plants were inoculated with transformed fungal culture using an art brush size 9. A WT inoculum was included as a positive control. The inoculation chamber was then covered with clear polythene sheet to create a humid condition. Plants were sprayed with water three times a day to maintain humidity levels of 80–90% for 72 h. Polythene sheets were removed from the inoculation chamber after 72 h. Disease severity index was scored according to Alvarado-Capo et al. (2003) with modification, a scale of 1–6, ^∗^6, and ^∗∗^6. (i.e., Score 1: No symptom, Score 2: Brown streak visible on underside of leaf but later visible on leaf upper surface as yellow streak; color changes progressively to brown black on upper leaf surface, Score 3: Enlarged stage 2 becomes longer as disease progresses, Score 4: Streak appears on underside as brown spot and black spot on upper leaf surface, Score 5: Elliptical spot totally black on the underside of the leaf surrounded by yellow halo, Score 6: Center of spot dries out turns gray, surrounded by a well defined margin and a bright yellow halo, Score ^∗^6: leaf dried/dead due to severe disease infection, Score ^∗∗^6: Leaf died due to normal aging processes, i.e., senescence).

### Determining Fungal Mycelium Growth and Symptom Development in Infected Plant Tissues

Leaf disk of approximately 2–3 cm of both inoculated and non-inoculated banana leaves were detached and soaked in 10 ml of absolute HCl for 20 min. The samples were then washed in sterile distilled water for 5 min and soaked twice in 100% ethanol for 1 h to remove chlorophyll. Finally, the leaf samples were rinsed in sterile distilled water for 5 min, dehydrated with 95% ethanol and stored at 4°C until ready for staining.

To observe fungal mycelium growth and development within banana leaf cells, the clear leaves were stained with LPCB for 30 min. Excess stains on leaf disk were blotted and fixed on slides. The slides were observed under COSLAB light microscope and picture was taken using digital camera MDCE- 5C (ISO 9001 Co) and analyzed using Optika Vision Lite 2.1 software.

### Detection of *P. fijiensis* in Plant Tissue

#### DNA Extraction

DNA was extracted from 1 g infected and non-infected banana leaf samples and pure culture of *P. fijiensis* as previously described (Mahuku, 2004). In order to remove the RNA contaminants, each of the DNA samples (50 μl) were treated with 1 μl of 10 mg/μl RNase A.

#### Real Time PCR Assay

Quantitation standard curve was used for *P. fijiensis* DNA detection and biomass estimation in infected, non-infected and pure fungal culture samples.

The amount of fungal genomic DNA detected in each of the sample analyzed was calculated using sample DNA Cycle threshold (*C*_t_) mean values, correlating to Y concentration values from the regression curve. The RT- PCR reaction mixture contained Maxima SYBR Green/ ROX qPCR Master mix (2x; Thermo Scientific), 300 nM primer (i.e., *β- tubulin*), 100 ng DNA in a total reaction volume of 12 μl. The program cycle was same as described in Expression assay above.

Each experiment has three technical replicates of three mutants and WT. The experiment was replicated twice.

### Statistical Analysis

The data obtained were analyzed using GenStat 7th edition statistical software package employing ANOVA to test significance difference and comparison of means.

## Results

### Preparation of RNAi Silencing Plasmid Construct

RNAi plasmid construct pKOIISD1 was developed based on plasmid pSD1 with dual convergent opposing RNA polymerase II promoters, PtrpC and Pgpd for filamentous fungi. A PCR amplified fragment of *PfHog1* was inserted at the EcoRI cloning site of pKOIISD1 (**Figure 1**). The RNAi construct was validated by confirming the presence of *PfHog1* gene through end point PCR using the primers (pSD15F and pSD13R) designed from the promoters region of pKOIISD1 plasmid construct. The insertion and orientation of insert *PfHog1* in pKOIISD1 was further checked by sequencing. The sense strand of *PfHog1* gene was driven by PtrpC promoter and its anti-sense strand was regulated by Pgpd promoter (**Figure 1**). The pSD1 vector system carrying opposing PtrpC and Pgpd promoters have got sense and anti-sense RNA of the target gene *PfHog1*. This system form dsRNA in the cell and are transcribed independently with control of two opposing RNA polymerase II promoters.

### *Agrobacterium tumefaciens*-Mediated Transformation (ATMT) of *P. fijiensis* and Molecular Analysis of Transformants

**FIGURE 2:**
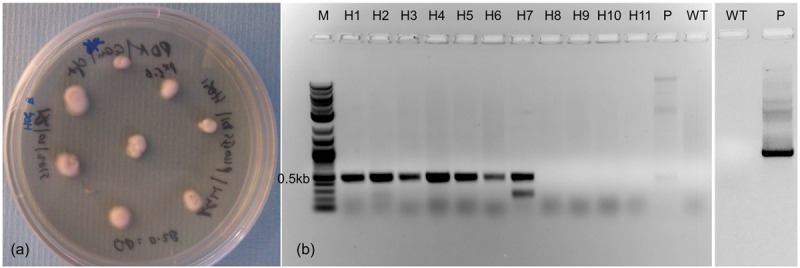
**Generation of *P. fijiensis* transformants through *Agrobacterium*-mediated transformation. (a)** Transformed mycelium on selective potato dextrose agar (PDA) medium containing geneticin. **(b)** Gel showing PCR amplification of *PfHog1* gene from genomic DNA of transformants of *P. fijiensis.* M, molecular marker; P, plasmid pKOIISD1-*PfHog1*; WT, control non-transformed *P. fijiensis*.

*ΔPfHog1* were generated by transformation of mycelial fragments using *A. tumefaciens* (**Figure 2a**). Neomycin phosphotransferase II (*nptII*) gene or (NeoR/KanR) was used as a selectable marker for the selection of transformed *P. fijiensis.* Agro-infected *P. fijiensis* were grown and selected on medium containing geneticin. Transformants *P. fijiensis* were validated by PCR analysis using *PfHog1* specific primers. The non-transformed *P. fijiensis* and pKOIISD1 plasmid were used as negative and positive controls for PCRs, respectively. The expected size (based on plasmid construct map) of amplified fragment of 0.5 kb was obtained from 6 of the 11 putative transformants of *P. fijiensis*, whereas two bands of 0.5 and 0.3 kb, respectively, were amplified from one of the putative transformants (**Figure 2b**). No amplification was observed in the non-transformed *P. fijiensis* that was used as negative control lacking the insert *PfHog1*.

### Expression Analysis of *PfHog1* Gene in Transformants of *P. fijiensis*

The expression of *PfHog1* gene was analyzed in the transformants of *P. fijiensis* by qRT-PCR using primers specific to WT *P. fijiensis* as a positive control. As low primer specificity can affect the accuracy of qRT-PCR assays, the specificity of the primers used in the qRT-PCR assays was verified by generating a linear regression curve using absolute quantitation. The *R* square (*R*^2^) and efficiency of the primer pair used for the amplification of *β-tubulin* from *P. fijiensis* was 0.99981 and 104.8%, respectively, whereas the *R*^2^ and efficiency of primer pair used for amplification of the *PfHog1* gene was 0.98 and 97.161%, respectively. A high linear correlation (*R*^2^> 0.91) suggest that similar experiments can be performed under similar conditions since *R*^2^ determines the precision and reproducibility of an experiment. A good qRT-PCR reaction should have an efficiency of 90–110%, which correspond to a slope between -3.58 and -3.10. While the *β- tubulin* primer had the lowest detection at *C*_t_ value of 20.257 and highest detection at *C*_t_ value of 33.363, and the *PfHog1* primer showed lowest detection at *C*_t_ value of 21.943 and highest detection at *C*_t_ value of 35.236. The samples with *C*_t_ values greater than 35 were considered as negative as this is in line with several q-PCR studies that used *C*_t_ value of 35 as the maximum limit for detection (Zhang et al., 2009).

**FIGURE 3:**
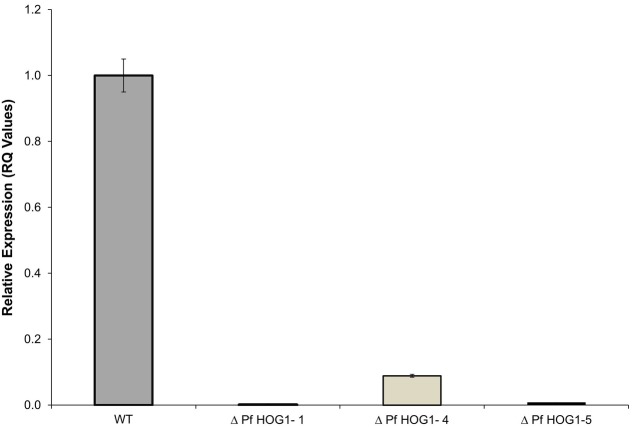
**Relative expression of *PfHog1* gene in the wild type (WT) and silenced mutants *P. fijiensis* Δ*PfHog1* Δ*(PfHog1-1*, Δ*PfHog1-4*, and Δ*PfHog1-5)*.** Three technical replicates of each mutant and WT control were used in each experiment. The experiment was repeated thrice and data are presented as Mean ± SE.

Expression level of *PfHog1* gene in the *P. fijiensis* transformants was performed using Relative (*C*_t_) Quantitation. Only three *P. fijiensis* mutants (i.e., *ΔPfHog1-1*, *ΔPfHog1-4*, and *ΔPfHog1-5*) were selected for expression in this study. The *β-tubulin* gene employed as a control of an endogenous housekeeping gene and *PfHog1* expression in wild-type non-transformed *P. fijiensis* as a reference control. Three biological replicate samples of transformed *P. fijiensis* were used. All the *P. fijiensis* mutants (i.e., *ΔPfHog1-1*, *ΔPfHog1-4*, and *ΔPfHog1-5*) used in the expression study had silenced *PfHog1* gene as shown in **Figure 3**. Level of *ΔPfHog1* gene expression (RQ value) was 0.00131 (0.131%) in *ΔPfHog1-1*, 0.08891 (8.89%) in *ΔPfHog1-4* and 0.00746 (0.746%) in *ΔPfHog1-5* as compared to RQ value of 1 (100%) in WT non-transformed *P. fijiensis*. This result confirmed that mycelium of *P. fijiensis* can be transformed through *A. tumefaciens.* About 63% of *ΔPfHog1* colonies randomly picked from the generated transformants where found positive.

### Response to Osmotic Stress in the *PfHog1*-Silenced Mutants of *P. fijiensis*

**FIGURE 4:**
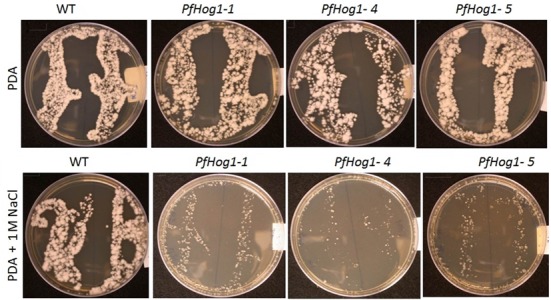
**Effect of silencing of *PfHog1* gene on osmotic stress regulation of *P. fijiensis*.** Growth and development of WT and Δ*Pf Hog1* mycelium on standard PDA and PDA supplemented with 1 M NaCl.

To study the role of *PfHog1* in osmotic stress, assays were performed to investigate tolerance of *ΔPfHog1* to high osmotic pressure on PDA medium supplemented with NaCl. Three independent mutants (i.e., Δ*PfHog1-1*, Δ*PfHog1-4*, and Δ*PfHog1-5*) were tested for osmotic stress regulation and similar results were obtained for all mutants tested. There was no difference in mycelial growth between the WT control and *ΔPfHog1* on standard PDA medium. However, the mycelial growth of the *ΔPfHog1* (i.e., Δ*PfHog1-1*, Δ*PfHog1-4*, and Δ*PfHog1-5*) on PDA medium supplemented with 1 M NaCl was much reduced by 55.46% (*p*-value = < 0.001, Lsd value = 1.832) at 14 days post-culture on the plates in comparison to WT (**Figure 4**). This study confirmed that *PfHog1* is critical for tolerance to osmotic stress, as 1 M NaCl significantly reduced mycelial growth of the Δ*PfHog1* compared to the WT.

### Virulence of the *PfHog1*-Silenced Mutant of *P. fijiensis* on the Banana Host

To determine the role of *PfHog1* in virulence of *P. fijiensis*, banana leaves of cultivar ‘Nakitembe’ were inoculated with mycelia of the Δ*PfHog1* mutants (Δ*PfHog1-1*, Δ*PfHog1-4*, and Δ*PfHog1-5*) and WT non-transformed *P. fijiensis*. The plants inoculated with the WT control strain developed symptoms of black Sigatoka disease in 9 to 10 days post-inoculation (dpi), whereas the development of disease symptoms in plants inoculated with the Δ*PfHog1* (Δ*PfHog1-1*, Δ*PfHog1-4*, and Δ*PfHog1-5*) was delayed and started appearing after 15 dpi.

**FIGURE 5:**
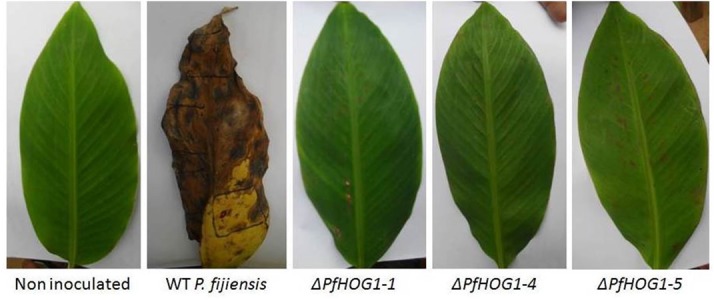
**Effect of silencing of *PfHog1* gene on the disease development in banana cultivar ‘Nakitembe’ at 45 days after inoculation with *P. fijiensis*.** Leaves inoculated with 10% rich medium containing 1% tween 20 acted as non-inoculated control. Leaves were inoculated with WT strain of *P. fijiensis* and three independent mutants Δ*Pf Hog1* (Δ*PfHog1-1*, Δ*PfHog1-4*, and Δ*PfHog1-5*).

Disease development following the initial appearance of symptoms on the leaves was also much faster in case of the WT control as compared to the Δ*PfHog1*, as shown by the higher levels of necrosis induced by the former at 45 dpi (**Figure 5**). Collectively, these results indicate that *PfHog1* is an important contributor to virulence of *P. fijiensis* on its host banana.

**FIGURE 6:**
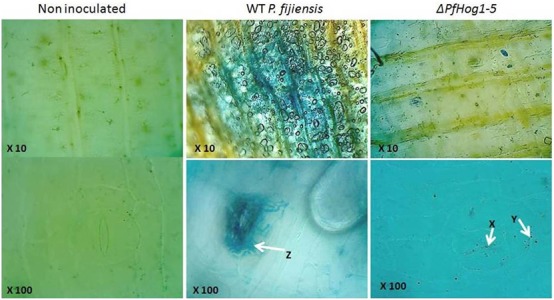
**Image showing mycelium growth and development in tissue of banana leaf stained with lacto phenol cotton blue.** Images were taken at 10X (upper panel) and 100X (lower panel) for non-inoculated banana leaf tissue, leaf inoculated with WT *P. fijiensis* and leaf inoculated with silenced mutant *P. fijiensis* (Δ*PfHog1-5*). Arrow Z is massive mycelium vegetative growth; arrow X, Y non-filamentous or invasive mycelium growth.

The vegetative growth and development of mycelium of *P. fijiensis* was monitored in banana leaf tissues. In this experiment, non-infected plant leaf tissues used as negative control was brushed with mixture of 10% rich medium and 1% tween 20 and leaf tissues from plants inoculated with the WT or the Δ*PfHog1* were stained with LPCB that binds to fungal chitin (**Figure 6**). As expected, no fungal growth was detectable in non-infected plant leaves and massive extracellular filamentous fungal growth was detected in leaves from plants inoculated with the WT *P. fijiensis*. Examination of plant leaves inoculated with the Δ*PfHog1* revealed substantially reduced fungal growth as compared to the WT control. No fungal filamentous growth was detected in plant leaves inoculated with the *ΔPfHog1*, indicating an impaired filamentous growth. Δ*PfHog1* remained in cyst or yeast like form within the extracellular space and failed to grow beyond the stomata.

### Detection and Quantification of *P. fijiensis* Biomass in Banana Leaves of Infected Plants

**FIGURE 7:**
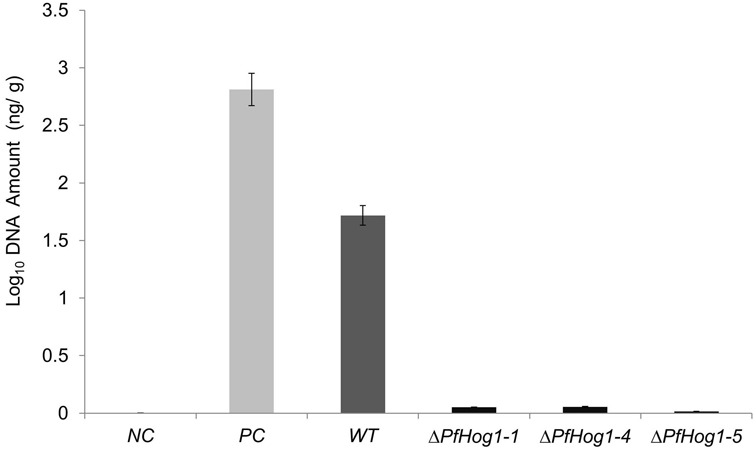
**The amount of DNA in different samples inoculated with WT and silenced mutant *P. fijiensis* (Δ*PfHog1*).** NC, Genomic DNA from banana leaf of non-inoculated plant; PC, DNA from pure *P. fijiensis* culture; WT, DNA from plant inoculated with WT *P. fijiensis*; Δ*PfHog1-1*, Δ*PfHog1-4*, and Δ*PfHog1-5*, DNA from plants inoculated with mutants. Each experiment has three technical replicates of three mutants and WT. The experiment was replicated twice. All the data are presented as Mean ± SE.

The relative growth and biomass of *P. fijiensis* was quantified in infected leaf tissue by qPCR. *C*_t_ values were plotted against the Log_10_ genomic DNA, to estimate DNA concentration (Y) from samples. A linear regression curve was generated where *Y* = -0.265X+6.0582. The lowest concentration (0.00062 ng/μl) was detected at *C*_t_ value of 33.363, meaning detection above *C*_t_ value 33.3 as a negative or noise from amplification. High DNA estimate was obtained in plant leaves inoculated with WT (1.718 ng/g) as compared to leaves inoculated with Δ*PfHog1* that had a low DNA estimate (Δ*L6* = 0.0517, Δ*L8* = 0.0555, Δ*L11* = 0.016 corresponding to Δ*PfHog1-1*, Δ*PfHog1-4*, and Δ*PfHog1-5* mutants, respectively) (**Table 1** and **Figure 7**). This is in agreement with the visual assessment of the virulence assays, where disease severity was lower in plants inoculated with Δ*PfHog1* and higher disease severity in plants inoculated with WT.

**Table 1:** Estimates of DNA and biomass of *P. fijiensis* in different banana samples inoculated with wild type (WT) and silenced mutants of *P. fijiensis* (Δ*PfHog1*).

Sample ID	Sample Details	*C*_t_ mean value (*SD*)	*C*_t_ mean (X10^1^)	DNA amount (ng/g)	Biomass estimate
NC	Negative control DNA from uninfected banana leaves	33.30 (0.73)	3.33	0.0008	7.62e-7
PC	Positive control DNA from pure culture of *P. fijiensis*	20.66 (0.70)	2.07	2.8119	0.0028
WT	DNA from banana leaves infected with WT *P. fijiensis*	21.42 (0.15)	2.14	1.718	0.0017
Δ L6	DNA from banana leaves infected with Δ*PfHog1-1*	26.81 (0.08)	2.68	0.00517	5.17e-5
Δ L8	DNA from banana leaves infected with Δ*PfHog1-4*	27.70 (0.17)	2.67	0.0555	5.55e-5
Δ L11	DNA from banana leaves infected with Δ*PfHog1-5*	28.61 (0.22)	2.86	0.016	1.6e-5


The result confirmed more vegetative growth of *P. fijiensis* in plants inoculated with the WT non-transformed control in comparison to Δ*PfHog1*. There was a positive correlation between the amount of DNA in a sample and *C*_t_ value. Lower *C*_t_ values corresponded to higher amount of DNA/biomass estimate and higher *C*_t_ values corresponded to lower amount of DNA/biomass estimate, as seen in **Table 1**. High relative growth and absolute biomass of *P. fijiensis* could only be detected in pure culture (positive control) and leaves inoculated with WT *P. fijiensis*. However, banana leaves inoculated with Δ*PfHog1* showed extremely low relative growth and absolute biomass of *P. fijiensis*. This result confirmed the observation on fungal growth in leaf tissues, the visual virulence assay assessment and leaf staining.

## Discussions

We explored the osmotic stress regulation and pathogenicity functions of *PfHog1* in *P. fijiensis* through silencing the targeted gene using RNAi technique. In this study, we used the plasmid vector pKOIISD1, specifically designed for RNAi silencing in fungi. The plasmid pKOIISD1 has *npt*II as selection marker. The *npt*II gene or (NeoR/KanR) is the most widely used selection marker. The gene codes for the aminoglycoside 3’-phosphotransferase enzyme, which inactivates by phosphorylation a range of aminoglycoside antibiotics such as kanamycin, neomycin, geneticin (G418), and paromomycin. In this study, transformed *P. fijiensis* were selected on medium supplemented with geneticin.

The plasmid pKOIISD1 is based on pSilent-Dual1 (pSD1) developed by Nguyen et al. (2008). The pSD1 is an RNA-silencing vector with a convergent dual promoter system that provides a high-throughput platform for functional genomics research in filamentous fungi. Involvement of several calcium signaling related genes in infection related development and pathogenicity of filamentous fungi *Magnaporthe oryzae* was demonstrated using pSD1 vector system (Nguyen et al., 2008). This provided new evidence that the pSD1 system is more feasible alternative for exploring gene function in fungi. Our study also confirms that pSD1 system is valuable frontline screening tool for gene function before its application in developing transgenic banana with resistance to fungal pathogens. Several post-transcriptional gene silencing studies have been described in protozoa *Plasmodium falciparum* (Malhotra et al., 2002; McRobert and McConkey, 2002), nematodes *Caenorhabditis elegans* (Brooks and Isaac, 2002), insects *Drosophila melanogaster* (Misquitta and Paterson, 1999; Hammond et al., 2001) and filamentous fungi *Neurospora crassa* (Cogoni et al., 1994). However, little is known about RNAi silencing in *P. fijiensis*. The ultimate goal of RNAi gene silencing in *P. fijiensis* is to understand biological function of genes, through phenotyping of silenced *P. fijiensis*.

Genetic transformation of *P. fijiensis* has been one of the major challenges in studying pathogenicity genes in this fungus. Several genetic transformation protocols have been developed for *P. fijiensis*, including using protoplasts (Balint-Kurti et al., 2001) and underwater shock waves with intact conidia (Escobar-Tovar et al., 2015). However, isolating protoplasts from *P. fijiensis* and stimulation of conidiation in this fungus under artificial conditions is very challenging. *P. fijiensis* is an economically important pathogen causing black sigatoka disease in banana globally. This pathogen has a number of pathogenicity strategies and the tools that are essential to understand molecular mechanisms are yet to be developed. ATMT have shown to be very efficient in *F. oxysporium* causing wilt disease in chickpea (Islam et al., 2012) and *Colletotrichum acutatum* causing anthracnose disease (Talhinhas et al., 2008), however, they used the conidia as compared to mycelium. In this study, a novel RNAi- mediated gene silencing method in *P. fijiensis* through ATMT of mycelial fragments was developed, which could be used to study function of genes in this fungus. This ATMT protocol helped us to identify mutants for osmolarity and pathogenicity testing, suggesting this technique is applicable for rapid generation of RNAi silenced mutants of *P. fijiensis*. Further, gene expression analysis for both WT and silenced mutant strains of *P. fijiensis*, showed that ATMT using mycelia provides the effective method for functional studies of pathogenicity genes.

Our results indicated that *PfHog1* is an important regulator of osmotic tolerance in *P. fijiensis*, similar to previous reports on other filamentous fungi and yeasts (Beever and Laracy, 1986; Schuller et al., 1994; Jin et al., 2012; Babazadeh et al., 2013; Wang et al., 2016). The growth and development of mycelia fragments of Δ*PfHog1* mutants were suppressed on PDA medium supplemented with 1 M NaCl; whereas WT *P. fijiensis* showed normal growth and development of mycelia. Suppression of mycelia growth and development is a result of osmotic stress. Both WT and silenced mutants showed normal mycelia growth and development on PDA medium without 1 M NaCl supplement. This demonstrates that *PfHog1* is involved in osmotic stress regulation. *Hog1* is well known for its role in osmotic stress regulation and is well studied in *Saccharomyces* spp. *Hog1* mutants of *S. cerevisiae* showed increased sensitivity to osmotic stress, whereby mutant cells treated with NaCl had reduced colony growth compared to WT (Schuller et al., 1994). *Hog1* has also been reported to play an important role in osmotic stress response in *Verticillium dahliae* (Wang et al., 2016), *C. glycerinogenes* (Ji et al., 2014) and *M. acridum* (Jin et al., 2012). NaCl is known to induce osmotic stress. Responses to salt stress are based on osmotic adjustments by osmolyte synthesis and cation transport systems for sodium exclusion (Mager and Siderius, 2002; Yancey, 2005). Therefore, exposing *P. fijiensis* mycelia in a hyper osmotic environment could lead to a rapid cell dehydration and arrest of cell growth. Under such condition, fungal cells enhance intracellular accumulation of osmolytes, and glycerol cell membranes.

The osmotic stress response seems to be essential during the infection process of *P. fijiensis* especially during penetration and colonization between plant cells. This is consistent with the observation that *Hog1* in *Verticillium dahliae* (*VdHog1*) regulates hyperosmotic stress response (Wang et al., 2016). Also the deletion of *OSM1* in *M. oryzae*, a homolog of *Hog1*, was shown to induce low resistance to osmotic stress, whereby hyperosmotic conditions resulted in morphological defects (Dixon et al., 1999). Similarly, *Bipolaris oryzae* exhibited reduced growth under hyperosmotic conditions upon deletion of *SRM1*, which is a homolog of *Hog1* (Moriwaki et al., 2007).

We demonstrated that silencing of *PfHog1* affects the development of symptoms of black Sigatoka disease. We observed that the banana plants inoculated with the WT *P. fijiensis* developed symptoms of black Sigatoka disease in 9–10 dpi, however, the plants inoculated with the mutant *P. fijiensis* showed mild disease symptoms after 15 dpi. Normally, black Sigatoka disease symptoms can appear as early as 10 dpi and could be much severe after 14 dpi under high humidity and high temperatures (Carlier et al., 2000; Marin et al., 2003).

In this study, we demonstrated that silencing of *PfHog1* compromised the ability of *P. fijiensis* to infect banana leaves, due to failing filamentous growth. Indeed Δ*PfHog1* failed to switch from yeast like form to filamentous growth in the extracellular space. These results conferred the previous studies in which silencing of *Hog1* in *Phytophthora sojae* impaired zoospore development and growth on both unwounded and wounded soybean (Li et al., 2010). Similarly disruption of *MgHog1* led to inability of *M. graminicola* to infect wheat leaves as a result of failed filamentous growth. In contrast pathogenicity was never compromised in the case of *Hog1* mutants of *Magnaporthe grisea* rice pathogen and *C. lagenarium* cucumber anthracnose pathogen (Dixon et al., 1999; Kojima et al., 2004). This clearly show that the role of HOG1 pathway in pathogenicity is species specific, meaning only fungal plant pathogens that need to change from yeast- like form to filamentous form are controlled by *Hog1* gene. Therefore, here we assigned *PfHog1* as a new pathogenicity factor of *P. fijiensis* since the silenced mutants are impaired in filamentous growth and are unable to penetrate or colonize the host plant tissues as clearly distinguished from the non-mutants which was able to colonize the extracellular space.

Detection and quantification of pathogen biomass *in planta* is very crucial in studying virulence, growth and development of fungal pathogens. In this study, a novel method of detection and quantification of *P. fijiensis in planta* was used. The qPCR assay developed here provides the most specific and sensitive technique to quantify *P. fijiensis* biomass in the banana leaves. *β-tubulin* was used as reference gene as it is widely and frequently used in qPCR experiments as a representative of constitutively expressed gene in fungi (Faguy and Doolittle, 1998; McKean et al., 2001; Einax and Voigt, 2003). *β-tubulin* is a good candidate to detection level of DNA and RNA of *P. fijiensis*. This study is the first report where qPCR was used to detect the relative growth and absolute biomass of *P. fijiensis* in virulence assay in banana. The amount of DNA detected in nanogram per gram can be converted to microgram per gram to get the biomass of fungal pathogen in the infected leaves. There is no tool at present being used to quantify *P. fijiensis* biomass in infected banana leaf tissues to determine disease severity. However, disease scores or proportion estimation are commonly being used to determine disease severity for selection of resistance and disease evaluation. This visual evaluation lacks accuracy and precision, where necrosis is low especially amongst the tolerant or resistance cultivars. Lack of well defined estimate compromises effort to understand and define gene regulation pathways and mechanism of resistance in host- pathogen interaction. In this experiment, we could use qPCR assay to quantify the fungal colonization in banana leaf tissue; meaning this study is suitable for quantification or estimation of disease severity of foliar fungal or bacterial pathogens due to its specificity and sensitivity. The results shown here is in line with other studies of relative growth and absolute biomass quantification of fungal pathogens such as *F. graminearum* in wheat kernel, *Botrytis cinerea* on grapes, *Magnaporthe oryzae* in rice and pathogenic fungi in susceptible genotypes of *Arabidopsis* (Qi and Yang, 2002; Brouwer et al., 2003; Diguta et al., 2010; Horevaj et al., 2011).

In summary, this study concluded that *Agrobacterium*-mediated transformation of *P. fijiensis* using mycelium fragments is an easy and efficient way to facilitate functional genomic studies in fungi. RNAi is a potential tool in exploring gene function in Ascomycete and could be an important genetic tool to develop disease resistant crop. This study reports an important effort in developing system for silencing and detecting function of vital fungal genes, which can confer resistance against fungal pathogen in crop plants. Here, we confirm *PfHog1* being important in osmotic stress regulation and pathogenicity of *P. fijiensis*. This study has provided insights for controlling black Sigatoka disease by developing transgenic banana using RNAi technology targeting pathogen gene *PfHog1*.

## Author Contributions

FO conceived and developed the research ideas, performed all experiments, collected data and written manuscript; GT shaped development of research, provided supervision and edited manuscript; L-HC contributed in vector design, gene cloning, and *Agrobacterium*-mediated transformation of fungus; BF and IS shaped development of research, provided technical guidance and research supervision and edited manuscript; JT supported gene expression studies, microscopy and edited manuscript; WT, JK, and CC provided supervision of research; and LT shaped development of research ideas, provided technical guidance and supervision for conducting research and writing manuscript, critically reviewed and edited manuscript.

## Conflict of Interest Statement

The authors declare that the research was conducted in the absence of any commercial or financial relationships that could be construed as a potential conflict of interest.
